# Multiple Renal Abscesses due to ESBL Extended-Spectrum Beta-Lactamase-Producing* Escherichia coli* Causing Acute Pyelonephritis and Bacteremia: A Case Report with a Good Outcome (No Drainage Required)

**DOI:** 10.1155/2016/9076813

**Published:** 2016-11-27

**Authors:** Abdalla Khalil, Musaad Qurash, Asem Saleh, Rasha Ali, Mohamed Elwakil

**Affiliations:** ^1^Internal Medicine Department, International Medical Center (IMC) Hospital, Jeddah, Saudi Arabia; ^2^Radiology Department, IMC Hospital, Jeddah, Saudi Arabia; ^3^Emergency Medicine Department, IMC Hospital, Jeddah, Saudi Arabia

## Abstract

Extended-spectrum beta-lactamase-producing Enterobacteriaceae urinary tract infections are challenging infections with increased mortality, morbidity, and failure of therapy. A 44-year-old Saudi male diabetic patient was seen at the ER of IMC Hospital with features of acute pyelonephritis: fever, burning urine, and left flank pain for three days. He was treated for cystitis at the Endocrine Clinic two weeks prior to his ER visit with nitrofurantoin and levofloxacin orally according to urine culture and sensitivity result. The patient was admitted, received IV meropenem, and continued to be febrile for three days. His urine and blood culture at ER grew the same ESBL-producing* E. coli* as in his urine culture from the Endocrine Clinic. His abdomen CT scan showed two left renal abscesses at the upper and middle poles. His temperature resolved on the fourth day of IV therapy. Intravenous meropenem was continued for 4 weeks after inserting PICC line and the patient was followed up by home healthcare. He was feeling better with occasional left flank pain and repeated abdomen CT scan showed complete resolution of both renal abscesses.

## 1. Introduction

Community and hospital acquired extended-spectrum beta-lactamase- (ESBL-) producing Enterobacteriaceae are prevalent worldwide [1].

ESBL-producing* Escherichia coli (E. coli) and Klebsiella pneumoniae* infections carry a higher mortality rate, higher risk of developing bacteremia, and failure of therapy compared to non-ESBL-producing isolates [2, 3].

Catheter acquired urinary tract infection is one of the most common healthcare acquired infections [4].

Prevalence surveys report that urinary catheter is the most common indwelling device, with 17.5% of the patients in European hospitals [4] and 23.6% in United States hospitals [5] having a catheter related infection.

Overall frequency of ESBLs from all isolates of Enterobacteriaceae in the United States was 16 percent in* K. pneumoniae*, 11.9 percent in* E. coli*, 10 percent in* K. oxytoca*, and 4.8 percent in* P. mirabilis* [6].

Prevalence is even higher in isolates from Asia, Latin America, and the Middle East [7], reaching 60 percent in* K. pneumoniae* isolates from Argentina and 48 percent in* E. coli* isolates from Mexico [8].

Renal abscess is a rare complication for complicated urinary tract infections due to Enterobacteriaceae.

We are presenting a case of multiple renal abscesses secondary to a complicated urinary tract infection (pyelonephritis and bacteremia) caused by ESBL* E. coli* in a diabetic morbidly obese middle-aged Saudi male patient. These abscesses responded to intravenous therapy with meropenem.

## 2. Case Report

A 45-year-old Saudi male known to have type II diabetes mellitus, mixed hyperlipidaemia, essential hypertension, and morbid obesity was seen at the Emergency Department with fever for three days associated with chills, dysuria, frequency, and incontinence of urine. He had repeated vomiting and left flank pain for one day.

He was seen at the Endocrine Clinic 2 weeks prior to his ER visit with burning urine and frequency and was treated for cystitis. His urine culture grew ESBL extended-spectrum beta-lactamase-producing* E. coli* >100.000 colonies and he received oral nitrofurantoin 100 mg 6-hourly and levofloxacin 500 mg orally once daily. His symptoms improved for 10 days and then recurred again.

He did not have a history of urethral catheterization, renal stone, or recurrent urinary tract infection. He denied history of illicit drug intake and his last admission to a hospital was two months ago for upper endoscopy.

On examination at the ER, his temperature was 38.3°C, his pulse rate was 110/minute, and his blood pressure was 110/70 mmHg. Respiratory rate was 18/minute, oxygen saturation was 96% on room air, and weight was 187 kg and height was 172 cm (BMI: 63.2 kg/m^2^).

He looked toxic and sweaty. He had tenderness at the left costovertebral angle and chest and heart exam were unremarkable.

His complete blood count showed increase in white blood cells, 12.2 × 10^9^/L, with neutrophils 78% and normal hemoglobin and platelets count. Serum creatinine was normal, 100 *μ*mol/L, and his electrolytes were normal too. Urinalysis showed positive nitrates, +3 leucocytes, white blood cells >50/HPF, red blood cells 30/HPF, and +2 ketones. Random blood glucose was 16 mmol/L, serum ketone bodies were negative, and C-reactive protein was raised, 29 mg/L (reference: 0–5 mg/L).

His previous urine culture at the Endocrine Clinic visit grew >100.000 colonies of Gram negative bacilli* E. coli* (morphology was done according to CDC algorithm) which were resistant to cefuroxime, cefepime, and ceftazidime (i.e., extended-spectrum beta-lactamase) and sensitive to meropenem, imipenem, gentamycin, and nitrofurantoin (culture sensitivity was done by MIC results obtained using automated Vitek 2 AST-GN69 and AST-XN06 cards).

His abdomen and pelvis ultrasound was unremarkable.

The patient was started on intravenous meropenem 1000 mg 8-hourly (first dose at the ER) and intravenous fluids. He was admitted to the medical unit where he continued to be febrile and had less pain at the left flank. Both his urine culture and blood culture taken at ER grew the same* E. coli* with the same sensitivity pattern as in urine culture collected at his previous Endocrine Clinic visit. He had two more repeated blood cultures after admission which came negative.

**Figure 1:**
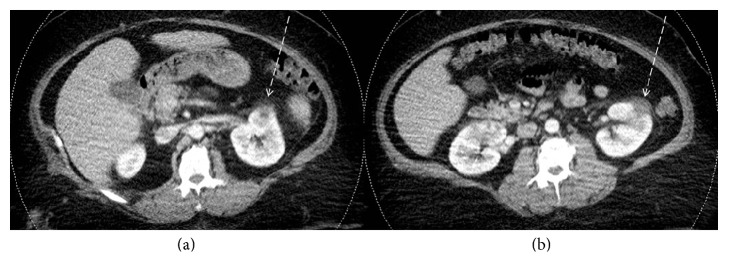
Abdomen CT scan with intravenous contrast on the third day of admission. It showed two left renal focal pyelonephritic changes in the upper and middle poles as well as the perinephric fat infiltration and thickening of Gerota's fascia. Also, scattered small para-aortic lymph nodes are noted. The first abscess in the upper pole is 2.5 × 2.3 cm in diameter (CT scan cut on (a)), and the other one in the middle pole is 2.6 × 1.8 cm in diameter (CT scan cut on (b)).

The patient had CT scan of the abdomen and pelvis on his third day of admission. It showed two left renal focal pyelonephritic changes in the upper and middle poles as well as the perinephric fat infiltration and thickening of Gerota's fascia. Also, scattered small para-aortic lymph nodes are noted. There is an abscess in the upper pole, 2.5 × 2.3 cm in diameter, and another in the middle pole, 2.6 × 1.8 cm in diameter (Figure 1).

The patient's temperature normalized on the fourth day on medication, and he started to feel better and the pain at the left flank nearly resolved. His white blood cells decreased to 6.0 × 10^9^/L and his C-reactive protein decreased to 10 mg/L (reference: 0–5 mg/L).

**Figure 2:**
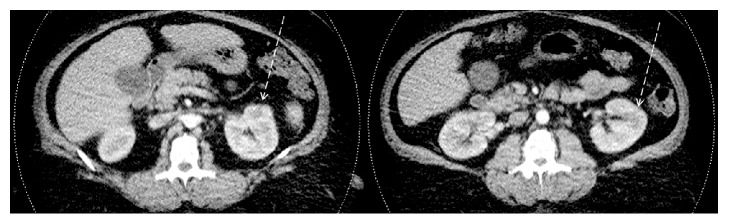
Findings. Abdomen CT scan with intravenous contrast after finishing 4 weeks of intravenous meropenem. It showed nearly complete resolution of the previously described abscesses with no perinephric fat stranding or collection.

Intravenous meropenem was continued after insertion of PICC (peripherally inserted central catheter) and the patient was educated on care of line, discharged, and followed up by a home healthcare team twice weekly. He was doing fine and afebrile during the 4-week course of antibiotic. He had occasional left flank pain that responded to simple analgesia paracetamol orally. His repeated compete blood count and C-reactive protein were in normal range. His repeated abdomen CT scan showed nearly complete resolution of the previously described abscesses with no perinephric fat stranding or collection (Figure 2).

## 3. Discussion

In Saudi Arabia, the prevalence of ESBLs Enterobacteriaceae isolates varies greatly in different regions.

It was shown to be 10.1% in the eastern province whereas in the central region it was 26.7% to 35.3% [9].

High level of carbapenem resistance had been observed in one study which was 20% [10]. In another study in the central region in Saudi Arabia, 57% of ESBL-producing* E. coli* isolates were of community origin [11].

In our electronic search (in Adult Medicine), we found only one case report of ESBL Gram negative bacilli causing renal abscesses [12].

In our present case, our patient was managed with nitrofurantoin and levofloxacin during his first visit to the Endocrine Clinic with urinary tract infection. Although the ESBL* E. coli* growing from urine culture was in vitro sensitive to nitrofurantoin, still carbapenem group of antibiotics intravenously is the drug of choice for the management of such case. His condition deteriorated after few days of partial improvement of his symptoms and he came to the ER with Gram negative ESBL (extended-spectrum beta-lactamase)* E. coli* bacteremia and failure of previous therapy.

Community acquired ESBL urinary tract infections are more challenging to manage than hospital acquired ones because treating physicians may not consider them on initiating antibiotic therapy and they will be detected later on after getting the final urine culture, that is, 48–72 hours.

We believe that we need more studies to assess the percentage of community acquired ESBL infections at other provinces of the kingdom.

## Abbreviations

ER: Emergency Room
HPF: High power field
PICC: Peripherally inserted central catheter
ESBL: Extended-spectrum beta-lactamase
IV: Intravenous.
